# An extract from date palm fruit (*Phoenix dactylifera*) acts as a co-agonist ligand for the nuclear receptor FXR and differentially modulates FXR target-gene expression *in vitro*

**DOI:** 10.1371/journal.pone.0190210

**Published:** 2018-01-02

**Authors:** Emilia Alfaro-Viquez, Brent F. Roling, Christian G. Krueger, Charlene J. Rainey, Jess D. Reed, Marie-Louise Ricketts

**Affiliations:** 1 Reed Research Group, Department of Animal Sciences, University of Wisconsin-Madison, Madison, WI, United States of America; 2 Department of Agriculture, Nutrition and Veterinary Sciences, University of Nevada, Reno, Reno, NV, United States of America; 3 Complete Phytochemical Solutions, Cambridge, WI, United States of America; 4 Date Research Institute, San Juan Capistrano, CA, United States of America; University of Texas at Austin Dell Medical School, UNITED STATES

## Abstract

Date palm fruit (*Phoenix dactylifera*) consumption reduces serum triglyceride levels in human subjects. The objective of this study was to prepare an extract from dates and determine whether it acts as a ligand for the farnesoid x receptor (FXR), a nuclear receptor important for maintaining triglyceride and cholesterol homeostasis. Freeze-dried extracts were isolated from California-grown dates (*Deglet Noor* and *Medjool*) from the 2014 and 2015 harvests, by means of liquid extraction and solid phase separation. Each date palm extract (DPE) was characterized via HPLC and MALDI-TOF mass spectrometry, and the procyanidin content was qualitatively determined. Extracts were tested to determine their ability to modulate nuclear receptor-mediated transactivation using transient transfection. The effect of DPE on FXR-target genes regulating bile acid absorption and transport was then assessed *in vitro*, in Caco-2 cells. Characterization reveals that DPE is a rich source of polyphenols including hydroxycinnamic acids, proanthocyanidins, and lipohilic polyphenols, and comprises 13% proanthocyanidins. Transactivation results show that DPE acts as a co-agonist ligand for both mouse and human FXR, wherein it activates bile acid-bound FXR greater than that seen with bile acid alone. Additionally, DPE alone activated a peroxisome proliferator activated receptor alpha (PPARα) chimera in a dose-dependent manner. Consistent with DPE as a co-agonist ligand for FXR, studies in Caco-2 cells reveal that co-incubation with bile acid, dose-dependently enhances the expression of fibroblast growth factor 19 (FGF19), compared to treatment with bile acid alone. In contrast, DPE inhibited bile acid-induced expression of ileal bile acid binding protein (IBABP). Our results demonstrate that DPE acts as a potent co-agonist ligand for FXR, and that it differentially regulates FXR-target gene expression *in vitro* in human intestinal cells. This study provides novel insight into a potential mechanism by which dates may exert a hypotriglyceridemic effect via FXR and modulation of bile acid homeostasis.

## Introduction

Date palm (*Phoenix dactylifera*) is one of the oldest cultivated trees and its fruit has been a dietary staple around the world for many centuries [1]. More than 2000 varieties of dates are grown worldwide, and date palm is an important crop in arid and semi-arid regions of the world including the Middle East, North Africa, parts of Central and South America, Southern Europe, India and Pakistan [2], as well as the Coachella Valley in California and Arizona in the U.S.

Dates are rich in carbohydrates, comprising 70–80% in the form of glucose and fructose. Date fruit also contains fiber, vitamins and minerals, as well as polyphenols, a class of bioactive compounds, especially phenolic acids [3, 4]. The carbohydrate, fiber and phenolic acid content depends on the date cultivar and ripening stage of the fruit [2] as well as environmental conditions [1]. Many date varieties were shown to contain *p*-coumaric acid, ferulic acid, sinapic acid and cinnamic acid derivatives, and isomers of 5-*O*-caffeoyl shikimic acid; while other date varieties were found to contain ferulic acid, caffeic acid, *p*-coumaric acid and *o*-coumaric acid, the concentration of which varied between 0.0606 to 0.1477 g/kg in dry dates [5, 6]. Flavonoids, also present in dates, are another important group of phenolic compounds that include proanthocyanidins, flavonoid glycosides and anthocyanins [3, 7].

Numerous beneficial health effects have long been associated with date fruit, including antioxidant, anti-mutagenic and anti-inflammatory activity, and protection of the gastric mucosa against damaging effects of stomach acid (reviewed in [1]). Hepatoprotective activity has also been linked to date fruit, including reduced alkaline phosphatase levels. These effects have been linked to the presence of anthocyanins, ferulic acid, caffeic acid, quercetin and proanthocyanidins [8–11]. Previous reports have also suggested that date fruit may provide protection against cardiovascular disease (CVD), the number one cause of death worldwide [12], by reducing hypertension, hypercholesterolemia, lipid oxidation, and by alleviating oxidative stress [1]. Dates were used for many centuries as an anti-hypertensive treatment in East Africa and the Middle East [1], however, the mechanistic understanding behind this observation was unknown until recently. Using *in vitro* studies, Braga and colleagues showed that date fruit could inhibit angiotensin converting enzyme (ACE) activity, an important target mediating reduced blood pressure both in pulmonary circulation and blood vessel endothelium [13]. This effect was found to be mediated via the phenolic compounds present within the fruit [13].

In addition to high cholesterol levels, elevated serum triglyceride levels are also an important risk factor that can contribute to the development of CVD [14]. Furthermore, metabolic syndrome (MetS), which is characterized by the co-presentation of several metabolic risk factors, including increased postprandial triglyceride levels and an increased ratio of low density lipoprotein to high density lipoprotein, affects ~23% of US adults [15], and significantly increases the risk of cardiovascular events such as heart disease and stroke [16].

Fruits and vegetables are rich sources of flavonoids, and have previously been shown to exert protective effects in human subjects against MetS-associated risk factors [17], as well as the prevention of CVD [18, 19]. Dietary proanthocyanidins, a class of flavonoids present in grapes, apples and red wine, were shown to attenuate risk factors associated with MetS [20–24]. Importantly with respect to the current study, consumption of 100 g dates per day (corresponding to ~7 dates) for four weeks was shown to reduce serum triglyceride levels in human subjects [25]. However, the underlying mechanism behind this observation remains unknown.

Nuclear receptors (NR’s) are critical players in whole body metabolic regulation [26–35] and are activated by numerous bioactive dietary compounds [36–46]. The farnesoid x receptor (FXR), a member of the nuclear receptor superfamily [47], is the major bile acid-responsive receptor important for maintaining bile acid, cholesterol and triglyceride homeostasis [48–54]. Previous studies using a luciferase reporter mouse model identified intestinal FXR as the critical regulator of bile acid signaling under normal physiological conditions [55]. As previously described in detail [42], intestinal bile acid absorption occurs via active transport in the distal ileum through the apical sodium-dependent bile acid transporter (Asbt) [56]. Once inside the cell, ileal bile acid-binding protein (Ibabp) [57], then binds to and transports the bile acids to the basolateral membrane, where they are then secreted, via the action of the heterodimeric organic solute transporters alpha and beta (Ostα/β) [58], into portal circulation for transpport to the liver. Fibroblast growth factor 15 (Fgf15; FGF19 in humans) expression is also induced by bile acid activation of intestinal FXR [59, 60], which leads to hormone-like fgf15/19 circulating to the liver where it binds to fibroblast growth factor receptor 4 (Fgfr4) complexed with β-Klotho. This activation stimulates the c-jun N-terminal kinase (Jnk) pathway, leading to suppression of *Cyp7a1*, which encodes cholesterol 7α-hydroxylase, the rate-limiting enzyme in the classical pathway for hepatic bile acid biosynthesis [59, 61–63]. When ileal FXR is activated by bile acids, *Asbt* is down-regulated [64], while *Ibabp*, *Ostα/β* and *Fgf15/19* are induced [59, 63, 65–67]. These FXR-mediated effects lead to decreased bile acid uptake from the intestinal lumen, increased bile acid transport into portal circulation, and reduced hepatic bile acid biosynthesis [59, 62, 63]. Essentially this critical FXR-mediated pathway along the gut-liver axis helps to regulate bile acid absorption, transport and biosynthesis and therefore the bile acid pool size and composition [60], as well as cholesterol and triglyceride homeostasis [52, 53, 68].

We previously showed that a grape seed procyanidin-rich extract (GSPE) is a bile acid-dependent co-agonist ligand for FXR [38], and that it reduces serum triglyceride and cholesterol levels via a pathway involving FXR, small heterodimer partner (SHP) and sterol regulatory element-binding protein 1c (SREBP1c) within the liver [37, 38]. More recently we showed that GSPE selectively modulates genes associated with bile acid absorption and transport in the intestine in an FXR-dependent manner, including *Asbt*, *Ibabp* and *Fgf15/19*, leading to decreased enterohepatic bile acid recirculation and increased fecal bile acid excretion [42]. These gene changes consequently lead to increased triglyceride catabolism, as well as hepatic bile acid and cholesterol biosynthesis, subsequently contributing to reduced serum cholesterol and triglyceride levels [42].

Based on the observation that date consumption reduced serum triglyceride levels in human subjects [25], combined with our previous studies elucidating the NR-mediated molecular mechanisms of action of GSPE [37, 38, 40–42], we prepared and characterized an extract from California-grown date palm fruit in order to test the hypothesis that dates contain bioactive compounds that could regulate FXR-mediated target-gene expression levels leading to the observed triglyceride-lowering effects in human subjects *in vivo*.

In order to determine whether date palm extract (DPE) exerts molecular regulatory effects via FXR, we systematically assessed the potential for DPE to transactivate FXR and a series of other NRs using transient transfection methodology and subsequently assessed the effect of DPE on FXR-target genes important for bile acid homeostasis *in vitro*, using Caco-2 cells. Herein, we now demonstrate that DPE, made from California-grown date palm, is a rich source of phenolic compounds; specifically, hydroxycinnamic acids, PACs, and lipophilic polyphenols. Furthermore, for the first time we show that DPE acts as a co-agonist ligand for FXR and that it differentially regulates FXR-target genes involved in bile acid homeostasis *in vitro*. Identification of an intestinally derived FXR-mediated effect may contribute a new molecular mechanism underlying the observed triglyceride-lowering effect observed following date palm consumption *in vivo* in human subjects. Furthermore, since dates contain polyphenols with a high degree of polymerization, they are likely not very bioavailable *in vi*vo. Therefore, the findings of this study suggest a novel mechanism by which poorly-bioavailable dietary bioactives from dates could exert a systematic anti-lipidemic effect without being absorbed into systemic circulation.

## Materials and methods

### Chemicals

Ethanol (200 proof) was obtained from Decon Labs (King of Prussia, PA, USA), while methanol and acetone (HPLC grade) were from Fisher Scientific (Fair Lawn, NJ, USA). Sephadex LH-20^TM^ was obtained from GE Healthcare (Uppsala, Sweden), Amberlite^TM^ FPX-66 ion exchange resin was from DOW (Philadelphia, PA, USA), 2,5-dihidroxybenzoic acid, butanol, and hydrochloric acid were obtained from Sigma Aldrich (Milwaukee, WI, USA). All other chemicals were obtained from Thermo Fisher Scientific unless otherwise stated.

### Preparation of date palm extract

California *Medjool* and *Deglet Noor* dates (*Phoenix dactylifera*) were provided by the California Date Commission (Indio, CA, USA). Dates were collected during the 2014 and 2015 harvest seasons. Each year, an extract was prepared by combining equal parts of *Medjool* and *Deglet Noor* dates, and homogenized to a fine powder by blending with liquid nitrogen and stored at –80°C until extraction.

The extraction procedure was adapted from our previously published reports [69, 70]. Briefly, 1 Kg of date powder was extracted using 2 L of 70% aqueous acetone (v/v) by sonication for 30 minutes, and filtered through a Whatman #43 filter paper. The filtered solution was then concentrated by rotary evaporation to a final volume of 500 mL. The extract was stored at 6°C overnight and then subjected to liquid chromatography. The aqueous extract was loaded onto four glass columns (1.5 cm I.D. x 15 cm length, Omnifit) packed with FPX-66 resin that was previously activated with ethanol and equilibrated with water. The resin bed was consecutively eluted with 200 mL of water and ethanol [71]. Four ethanolic fractions were collected and pooled together and concentrated by rotary evaporation to dryness and re-solubilized in water to a final volume of 200 mL. The aqueous extract was stored at -20°C for 24 h, and lyophilized to obtain a powdered date palm extract (DPE). Approximately one gram of DPE was obtained from one kilogram of dates.

### Characterization of date palm extract

#### Total proanthocyanidin determination

The total proanthocyanidin (PAC) content in DPE was determined by an adjusted acid-butanol method [72]. Briefly, 5 mL of acid butanol reagent (95% v/v hydrochloric acid in n-butanol) was added to 5 mg of DPE, incubated at 100°C for 60 min, followed by rapid cooling to room temperature in an ice bath. The absorbance was measured at 550 nm using a microplate reader (Molecular Devices SpectraMax Plus 384). A stock solution of purified PAC (63.13 mg/mL) was used to generate external standard curves [69]. The purified PAC was obtained by acetone extraction of cranberry fruit and subsequent liquid chromatography using Sephadex LH-20. The purified PAC extract was characterized by the Folin-Ciocalteu method, HPLC-DAD, formaldehyde-HCl precipitation, elemental analysis, MALDI-TOF MS, and matrix-assisted laser desorption/ionization–Fourier transformation ion cyclotron resonance mass spectrometry (MALDI-FT-ICR MS). The purity of the PAC was estimated to be 99.0 ± 1.3% with a mean degree of polymerization between 3 and 26. Results are expressed as milligrams of PAC equivalents per gram of DPE (mg PACs/g). Each experiment was performed in triplicate.

#### Isolation and characterization of DPE-PACs

The extract obtained previously was further separated using a glass column (2.5 cm I.D. x 5 cm length, Flex-Column, Kimble) packed with Sephadex LH-20^TM^ resin, which was previously swollen and equilibrated in water. Two hundred milligrams of the extract was solubilized in 5 mL of water and loaded onto the column, and fractions were consecutively eluted with 75 mL of water, ethanol, ethanol:methanol (1:1 v/v), and water:acetone (2:8 v/v). The water:acetone (2:8 v/v) fraction, which contains approximately 67 mg of DPE-PACs, was concentrated by rotary evaporation to dryness and re-solubilized in 5 mL of methanol [69]. DPE-PACs were then characterized by RP-HPLC-DAD and MALDI-TOF MS, as described below.

#### RP-HPLC-DAD analysis

Reversed-phase high performance liquid chromatography with diode-array detector (RP-HPLC-DAD) was performed in order to further characterize the chemical composition of DPE. Fifty milligrams of DPE was dissolved in 1 mL of water and filtered through a 0.45 μm nylon syringe filter. Then a 20 μL aliquot of the DPE solution was injected into a Waters Spherisorb ODS2 RP-18 column (25 μm 4.6 × 250 cm). The solvents used for elution were 0.1% trifluoroacetic acid in water (solvent A) and methanol (solvent B). The HPLC system consisted of a Waters automated gradient controller, two Waters 501 HPLC pumps, and a Rheodyne 9010 manual injector. The flow rate was maintained at 2 mL/min, and elution was monitored by a Waters 996 diode array detector using Waters Empower software for collecting and analyzing three dimensional chromatograms.

#### MALDI-TOF-MS analysis

Mass spectra for DPE samples were collected on a Bruker Microflex LRF^TM^ MALDI TOF mass spectrometer (Billerica, MA, USA). All analyses were performed in positive reflectron mode. Spectra were the sum of different locations in each well, accumulating a total of 2000 shots with deflection set at 500 Da. A 10 μL aliquot of each sample was mixed with 10 μL of 2,5-dihydroxybenzoic acid (DHB, 50 mg/mL in ethanol), and 1 μL was spotted on the MALDI-TOF MS stainless steel target. FlexControl and FlexAnalysis (Bruker Daltonik GmbH, Bremen, Germany, version 3.0) were used for data acquisition and data processing, respectively. mMass (version 5.5.0) was used for spectra analysis and absolute intensities (*ai*) [73]. After acquisition, the MALDI-TOF spectra were subjected to the deconvolution method based on the relative intensity of the PAC isotope patterns, in order to calculate the ratio of “A-type” to “B-type” bonds present within DPE-PACs.

#### Deconvolution method

An understanding of the natural abundance of carbon, hydrogen, and oxygen isotopes within PAC oligomers previously allowed us to develop a method to determine the ratios of A- to B-type interflavan bonds in cranberry PAC [73]. A series of PAC which vary only in the ratio of A- to B-type interflavan bonds produces mass spectra with overlapping isotope patterns for each individual oligomer. Spectrum obtained for DPE were therefore subject to analyses of deconvolution according to previously described methods [73, 74]. Data were excluded from the analysis when one of the peaks included in the deconvolution of isotope pattern had a signal/noise ratio of less than 3.0. Cumulative A-type interflavan bond distribution was calculated from the deconvolution data as the sum of all percentage distributions showing at least one A-type interflavan bond at a degree of polymerization.

### Transient transfection

The Gal4 DNA-binding domain-receptor ligand-binding domain chimeras [75], full-length human FXR [51], human FXR ΔAF2 mutant, human FXR W469A mutant [76], full-length murine FXR, and the Δ9C murine FXR mutant [77] have all been reported previously [45].

CV-1 cells were purchased from ATCC® and were used between passage numbers 10–30 for these studies. Cells were maintained in DMEM supplemented with 10% fetal bovine serum (FBS) and 1% L-glutamine, and cultured at 37°C and 5% CO_2_. Transient transfection was performed using the calcium phosphate precipitation method as previously reported [45]. Cells were assayed for luciferase (Promega) activities 24 h after addition of ligands, and reporter expression was normalized to β-galactosidase (Applied Biosystems, Chicago, IL). The doses of DPE used in these *in vitro* assays are consistent with those used previously to test GSPE [38, 42]. Furthermore, from 100 g of dates we have prepared 100 mg of extract, which likely contains less than the total amount of polyphenols actually present within 100 g of dates. Consequently, based on the fact that the human small intestine fluid volume following a meal is reported to be 20–156 mL [78], a 100 mg dose of extract would correlate to between 641 mg/L and 5 g/L physiologically *in vivo*. Therefore, the doses used in vitro in this study are physiologically relevant and it is feasible that a higher level of polyphenols would be available in the gut following consumption of 50–100 g of whole dates. Data represents the mean ± SEM for the fold change relative to the DMSO control. Similar results were obtained from at least three independent experiments, performed in triplicate.

### Caco-2 cell culture

Caco-2 cells (HTB-37^TM^) were purchased from ATCC® and used between passage numbers 5–10 for these studies. Cells were maintained in 10 cm Corning cell culture dishes in Dulbecco’s Modified Eagles medium (DMEM) supplemented with 20% FBS, 1% L-glutamine, and cultured at 37°C and 5% CO_2_, as previously described. Caco-2 cells were chosen as an *in vitro* model because they express FXR 10 days post-confluence and are an established model commonly used to assess effects on FXR-target genes, as previously reported [42, 75, 79]. As previously described in detail [42], once the cells reached confluence, they were sub-cultured into 6 well plates, at 1 x 10^6^ cells per well for subsequent experiments. The cells were allowed to reach confluence and grown an additional 10-days post-confluence, with replacement of fresh media every 48 hours. Cells were then grown for an additional 24 hours, after which the media was removed and replaced with DMEM supplemented with 1% L-glutamine and 0.5% charcoal-stripped FBS. As previously reported, a lower concentration of FBS was used to minimize the effect of bile salts commonly found in FBS [42, 79], which may otherwise cause interference when assessing the effects of DPE. After 24 hours media was replaced, cells were treated for 12 hours with either DMSO, 100 μM chenodeoxycholic acid (CDCA) (Sigma-Aldrich), DPE (20, 50 or 100 mg/L), or a combination of both CDCA and DPE in DMEM supplemented with 1% L-glutamine and 0.5% charcoal-stripped FBS. Similar results were obtained for both the 2014 and 2015 extracts, analyzed in quadruplicate.

### Gene expression analysis

Total RNA was extracted from Caco-2 cells using TRIzol (Life Technologies) according to the manufacturer’s instructions. Complimentary DNA (cDNA) was reverse transcribed using superscript III reverse transcriptase (Life Technologies), and real-time quantitative polymerase chain reaction (qPCR) was used to determine gene expression changes. qPCR was performed using a CFX96 Real-Time System (BioRad). Forward and reverse primers were designed using OligoSys (Sigma-Aldrich) and purchased from Integrated DNA Technologies. Expression of *GUSB* was used as the endogenous control. Primer and probe sequences can be provided upon request. Data represents the mean ± SEM for the percent fold change relative to the DMSO control (n = 4).

### Statistical analysis

One-way analysis of variance (ANOVA) with Tukey’s multiple comparison tests was used to detect significant differences between treatments. Differences were considered statistically significant at *p*<0.05. All statistical analyses were performed using GraphPad Prism version 6.07 for Windows, GraphPad Software (San Diego, CA).

## Results

### Date palm extract is a rich source of phenolic compounds

**Fig 1:**
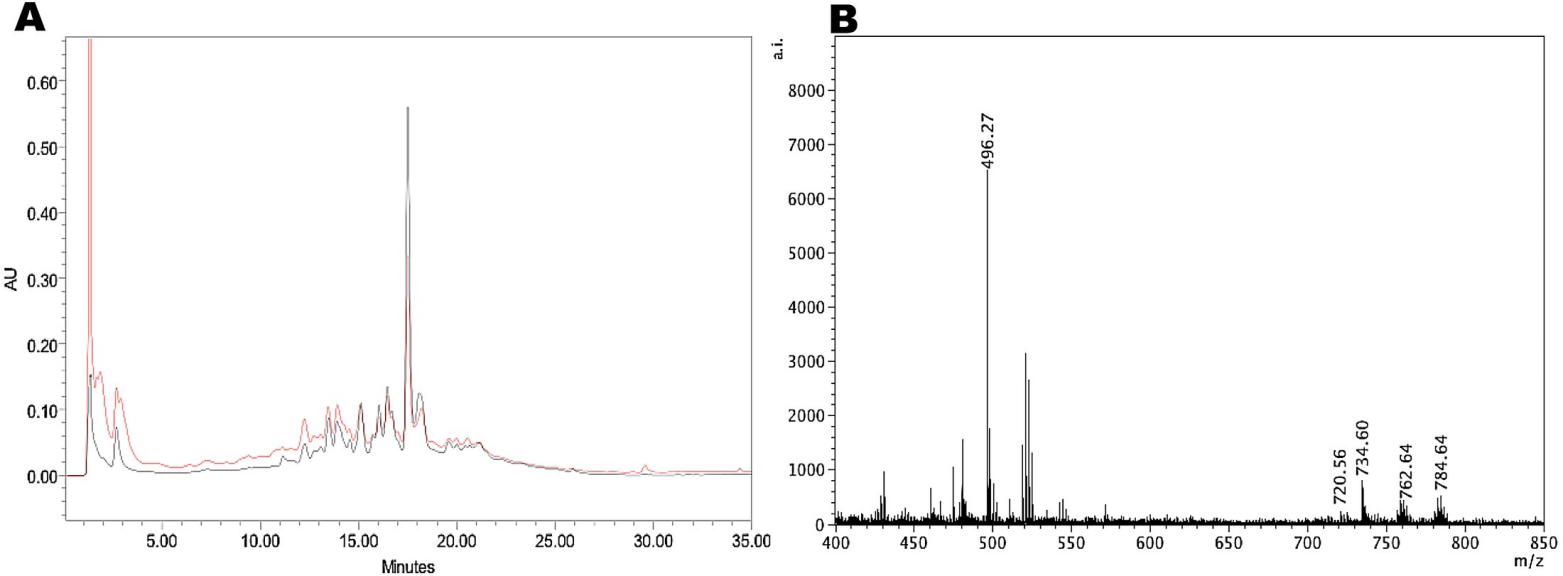
RP-HPLC-DAD and MALDI-TOF mass spec analysis of date palm extract. **A**. RP-HPLC-DAD chromatograms for DPE at 280 nm (red) and 320 nm (black); and **B**. Positive reflectron mode MALDI-TOF MS spectra showing a series of peaks at m/z 734 (Δ238amu), m/z 762 (Δ266amu) and m/z 784 (Δ288amu) that may correspond to long-chain ω–hydroxyfatty acids (C16, C18, C20) esterified to the trans- Feruloyloxy octadecanoic acid. AI: absolute intensities; AU: absorbance units.

Extraction of *Deglet Noor* and *Medjool* dates produced a powdered date palm extract (DPE), which has a total proanthocyanidin (PAC) content of 13% (131.28 mg PACs/g of DPE), as assessed via the acid butanol assay. The PAC reference standard was derived from cranberries as previously described by Feliciano et al., [69] and the quantification of date PACs is based on the assumption that the stoichiometry of the auto-oxidation reaction is similar between cranberry and date proanthocyanidins. The HPLC chromatogram of DPE represented in **Fig 1A** shows that the predominant polyphenols present within the extracts have spectral characteristics indicative of hydroxycinnamic acids, as suggested by its maximum UV-Visible absorbance in the range of 320–330 nm (shown in red). The extracts also have spectral characteristics indicative of the presence of PACs; showing a poorly eluting ‘hump’ rather than well-defined individual peaks at 280 nm absorbance (shown in black) [80, 81]. MALDI-TOF MS analysis of DPE is shown in **Fig 1B**. Polyphenol compounds were tentatively identified by comparison of their UV-Vis absorption spectrum and the MS data with compounds previously reported in the literature [7, 82]. The results suggest that hydroxycinnamic acids are the predominant polyphenol compounds present in DPE, with the data indicating the presence of caffeic, *p*-coumaric and ferulic acids, as well as cinnamic acid derivatives.

Positive reflectron mode MALDI-TOF MS analysis of DPE, also exhibited a series of compounds which appear to be oligomeric in nature with repeatable extension units (**Fig 1B**). The spectra shows a series of peaks at m/z 734 (Δ238 amu), m/z 762 (Δ266 amu) and m/z 784 (Δ288 amu) that can be assigned to suberin compounds, specifically an ester of ferulic acid with long-chain ω-hydroxyfatty acids (C16, C18, C20) [83]. Many of the compounds have mass differences of Δ2 amu (for example; 756, 758, and 760), suggesting variations in the degree of unsaturation. Overall, the pattern of peaks suggests the presence of a series of ω-hydroxyfatty acids with varying degrees of unsaturation.

**Fig 2:**
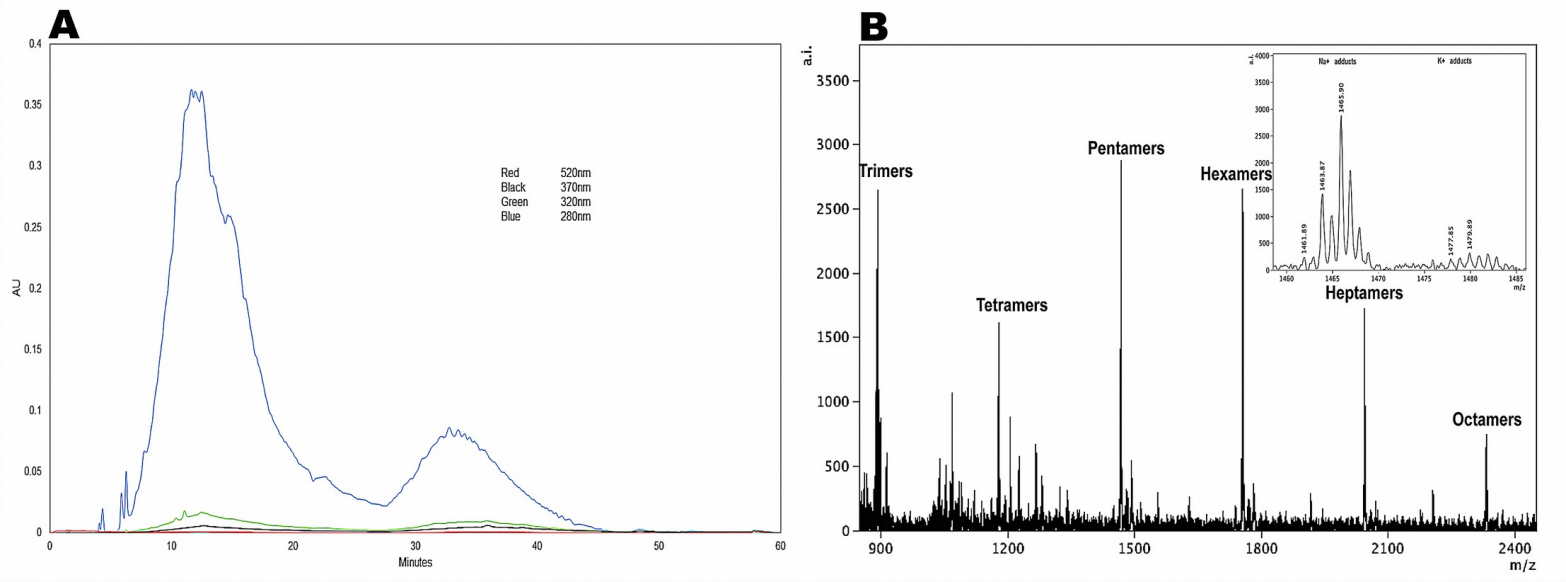
RP-HPLC-DAD and MALDI-TOF mass spec analysis of a PAC-enriched DPE fraction. **A.** RP-HPLC-DAD chromatogram for the PAC-enriched fraction of DPE following the Sephadex LH-20 column (DPE-PAC) collected at different wavelengths (Blue: 280, Green: 320, Black: 370, and Red: 520 nm); and **B.** MALDI-TOF MS spectra of DPE-PAC in positive reflectron mode, showing a series of PACs ranging from trimers to octamers. The Insert is a spectrum showing the overlapping isotope pattern for a DPE-PAC pentamer with Na^+^ and K^+^ adducts. AI: absolute intensities; AU: absorbance units.

Prior to characterization of the PACs present within DPE, the extract was first fractionated using a Sephadex LH20 column, thereby providing an enriched DPE-PAC fraction. As shown in **Fig 2A**, the RP-HPLC-DAD chromatogram for DPE-PACs at 280 nm (blue) shows two broad unresolved humps, which is often associated with the structural heterogeneity of PAC oligomers. Additionally, no other characteristic polyphenolic compounds, such as hydroxycinnamic acids (320 nm; green), flavonols (370 nm; black), and anthocyanins (520 nm; red) were observed following RP-HPLC-DAD of the DPE-PAC samples [69, 80].

MALDI-TOF MS is considered a more suitable technique for the analysis of PACs, which exhibit greater structural heterogeneity [84, 85]. MALDI-TOF MS produces only a singly charged molecular ion for each parent molecule and allows detection of high masses with precision. Structural variation of PACs, number of catechin/epicatechin monomers (Δ288 Da), galloyl residues (Δ156 Da), hydroxyl substitutions (Δ16 Da) and differences in A-type vs B-type interflavan bonds (Δ2 Da) can be predicted with MALDI-TOF MS. DPE-PACs were identified to contain a series of polyflavan-3-ol oligomers based on a repeating unit structure of (epi)catechin with one or more B-type interflavanyl linkages present in the oligomer [74].

MALDI-TOF mass spectral analysis of the DPE-PAC enriched fraction indicates that the degree of polymerization ranges from 3–8 (catechin/epicatechin) units (**Fig 2B**). Furthermore, MALDI-TOF mass spectral analysis and the HPLC chromatographic data suggest that the DPE-PAC fraction obtained from chromatographic separation using the Sephadex LH-20 resin facilitated the production of a DPE-PAC isolate free from other monomeric polyphenols, such as hydroxycinnamic acids, anthocyanins, and flavonols. PACs associate with sodium [M + Na]^+^ and potassium [M + K]^+^ forming alkali metal adducts [85], thereby splitting the signal unevenly, as shown in **Fig 2B**. The estimated percentages were therefore obtained by deconvolution of Na^+^ and K^+^ adducts as previously reported [73].

**Fig 3:**
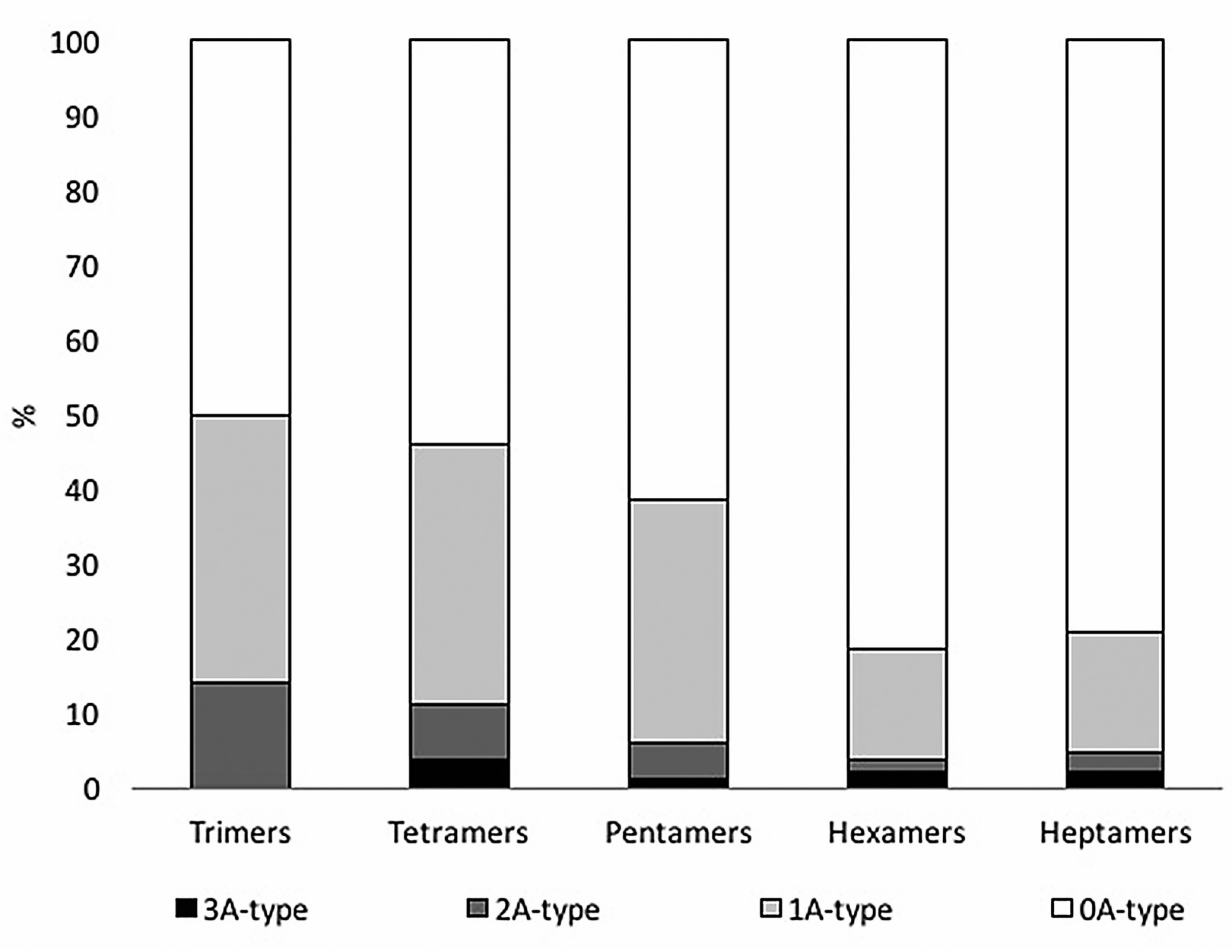
Percentage of A- and B-type interflavan bonds present in DPE-PAC. The percentage of A- and B-type bonds was calculated using matrix algebra for overlapping isotopic peaks after MALDI-TOF MS.

Using the deconvolution method based on relative intensity (ri) of the isotope pattern and absolute intensity (ai) for the analysis of the MALDI-TOF spectra [73], the ratio of A- to B-type interflavan bonds can be calculated, as shown in **Fig 3**. In the DPE-PAC spectra, the peaks with the highest absolute intensity correspond to 0 “A-type” bonds. The median values of the deconvolution method show that 67% of DPE-PACs are “B-type”. The results indicate that DPE-PACs have a similar distribution of A- and B- type interflavan bonds at lower degrees of polymerization (DP = 3). However, at higher degrees of polymerization (4 to 10) DPE-PACs reach high distributions of B-type interflavan bonds (> 90%).

### Date palm extract acts as a co-agonist ligand for FXR

**Fig 4:**
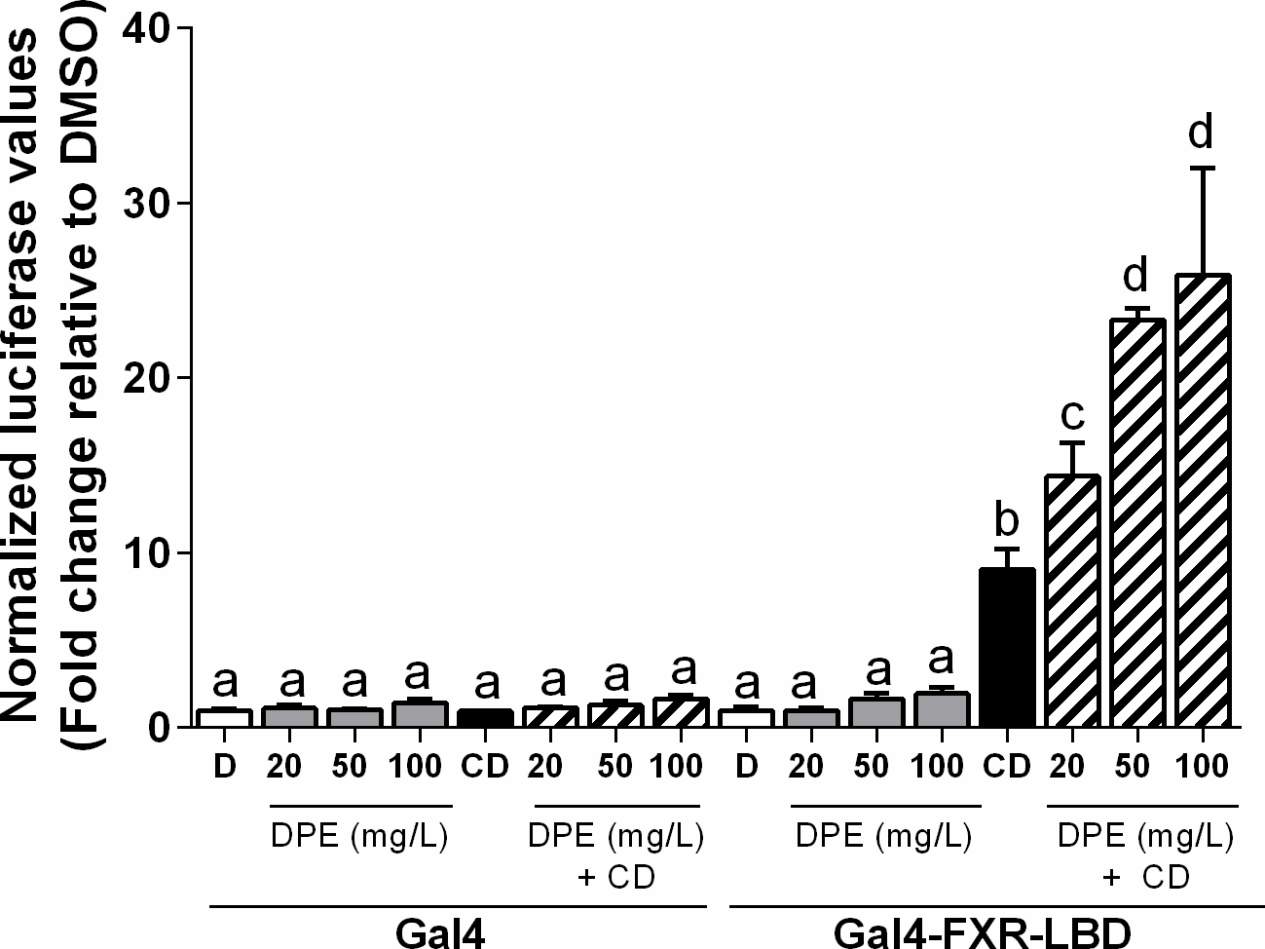
Determination of the ability of DPE to transactivate the ligand binding domain of the nuclear receptor FXR *in vitro*. CV-1 cells were co-transfected with a Gal4 luciferase reporter and a Gal4 DNA-binding domain construct or a chimera in which the Gal4 DNA-binding domain is fused to the ligand-binding domain of FXR. The cells were treated with a known receptor-specific agonist, 100 μM chenodeoxycholic acid (CD) or DPE (mg/L). Results are expressed as normalized luciferase activity relative to DMSO (set at 1) (mean ± SEM). Statistical differences are represented by letters. Bars with the same superscript letter are not significantly different from each other.

Following extraction and characterization, we next wanted to test the ability of DPE to transactivate the nuclear receptor FXR. We therefore used transient transfection methodology to investigate the effect of DPE on several nuclear receptors. As previously described [45], in the Gal4-based transactivation assay, the ligand-binding domains (LBD) of different nuclear receptors are fused to the Gal4 DNA binding domain, and effects on expression directed by a Gal4-dependent reporter plasmid are tested [45]. As expected, the established ligand for FXR, chenodeoxycholic acid (CDCA), significantly transactivated Gal4-mouseFXR-LBD (*p*<0.01) (**Fig 4**). DPE alone did not significantly enhance transactivation, however, it did dose-dependently enhance transactivation of bile acid-bound FXR, compared to CDCA alone (*p*<0.0001) (**Fig 4**). No such effects were observed with just the Gal4-DNA binding domain alone.

**Fig 5:**
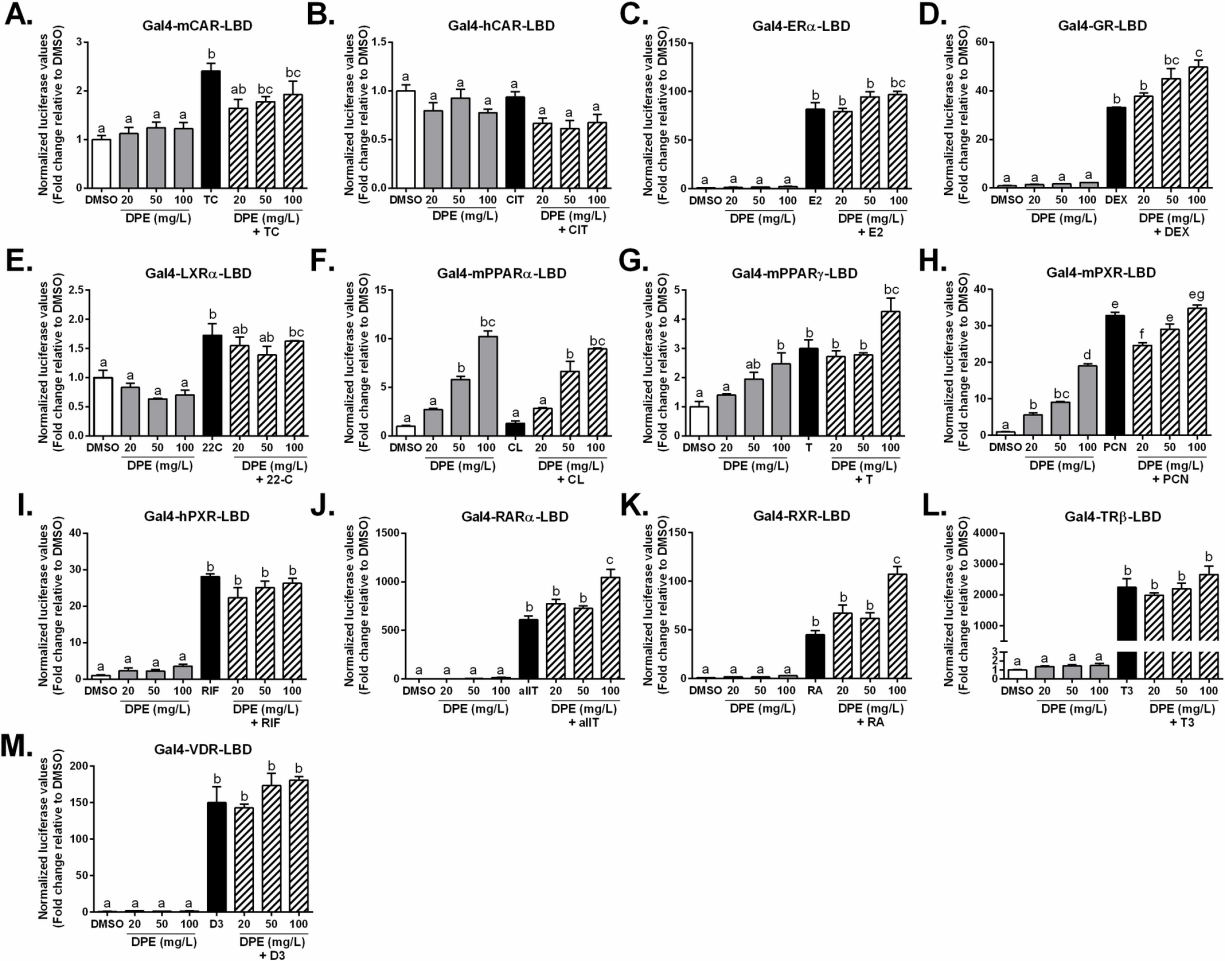
Determination of the ability of DPE to interact with a range of nuclear receptor ligand binding domains *in vitro*. CV-1 cells were co-transfected with a Gal4 luciferase reporter and a series of chimeras in which the Gal4 DNA-binding domain is fused to the indicated nuclear receptor ligand-binding domain. The cells were treated with a known receptor-specific agonist or DPE (mg/L). Results are expressed as normalized luciferase activity relative to DMSO (set at 1) (mean ± SEM). The ligands used were as follows: **A.** Mouse constitutive androstane receptor (mCAR): 250 nM 1,4-bis[2-(3,5-dichloropyridyloxy)] benzene (TC); **B.** Human CAR: 10 μM CITCO (CIT); **C.** Estrogen receptor alpha (ERα): 1 μM Estradiol (E2); **D.** Glucocorticoid receptor (GR): 100 nM Dexamethasone (DEX); **E.** Liver x receptor alpha (LXRα): 10 μM 22-hydroxycholesterol (22-C); **F.** Peroxisome proliferator-activated receptor (PPARα): 1 μM Clofibrate (CL); **G.** PPARϒ: 1 μM Troglitazone (T); **H.** Mouse pregnane x receptor (mPXR): 10 μM pregnane 16α-carbonitrile (PCN); **I.** human PXR: 10 μM Rifampicin (RIF); **J.** Retinoic acid receptor alpha(RARα): 1 μM all-*trans* retinoic acid (allT); **K.** Retinoid x receptor (RXR): 1 μM 9-*cis*-retinoic acid (RA); **L.** Thyroid hormone receptor beta (TRβ): 1 μM thyroid hormone (T3); and M. Vitamin D receptor (VDR): 100 nM 1α,25-dihydroxyvitamin D3 (D3). Statistical differences are represented by letters. Bars with the same superscript letter are not significantly different from each other.

In order to determine the specificity of DPE for FXR, several Gal4-NR-LBD constructs were then tested. As shown in **Fig 5A and 5B**, neither mouse nor human constitutive androstane receptor (CAR) were transactivated by DPE alone. Also, no co-agonistic effects of DPE were seen in the presence of their respective ligands, although it appears that co-incubation of TCPOBP with DPE had a slight inhibitory effect on mCAR, this did not reach significance.

No effects were observed for DPE with the estrogen receptor alpha (ERα) chimera either alone or in combination with estrogen (E2) (**Fig 5C**), whereas transactivation of the glucocorticoid receptor (GR) chimera does appear to be enhanced dose-dependently by DPE in the presence of dexamethasone (DEX), compared to DEX alone (**Fig 5D**). Transactivation of the liver x receptor alpha (LXRα) chimera was significantly enhanced by its ligand, 22-hydroxycholesterol, but was not affected by DPE alone or in combination with the ligand (**Fig 5E**). Interestingly, DPE did dose dependently enhance transactivation of the peroxisome proliferator activated receptor alpha (PPARα) chimera (50 and 100 mg/L DPE: *p*<0.0001). Unfortunately, no significant transactivation was observed with the PPARα ligand, clofibrate, in this study, however, no differences were observed between DPE alone and DPE plus clofibrate, indicating that DPE acts as an agonist ligand for mouse PPARα (**Fig 5F**). The highest concentration of DPE tested (100 mg/L) did enhance transactivation of the mouse PPAR_ϒ_ chimera (*p*<0.05) (**Fig 5G**), but did not exert any additive effect in the presence of the ligand troglitazone, compared to troglitazone alone.

**Fig 6:**
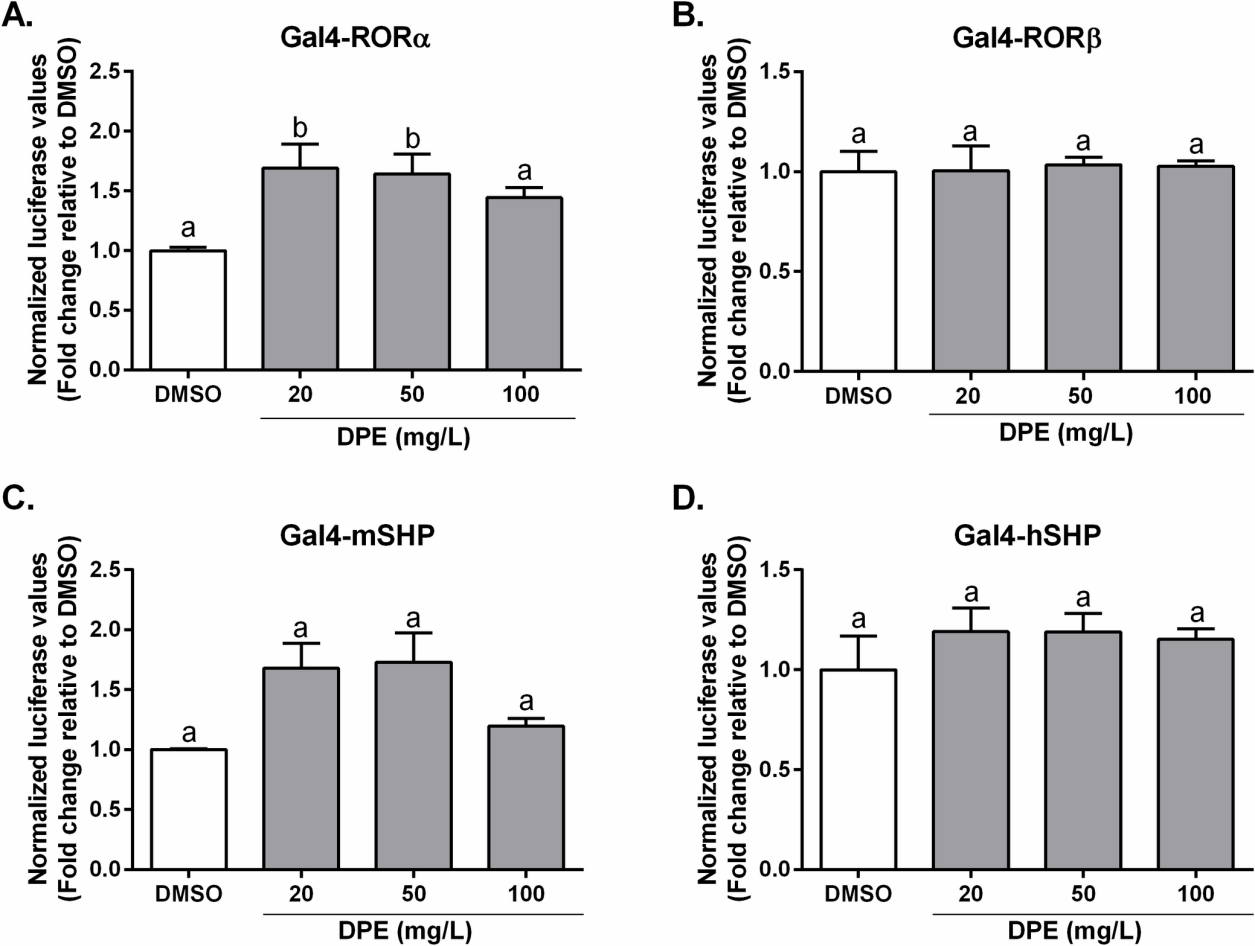
Determination of the ability of DPE to transactivate a range of nuclear receptors *in vitro*. CV-1 cells were co-transfected with a Gal4 luciferase reporter and a series of chimeras in which the Gal4 DNA-binding domain is fused to the indicated nuclear receptor: **A.** RAR-related orphan receptor alpha (RORα), **B.** RORβ, **C.** mouse small heterodimer partner (SHP), and **D.** human SHP. The cells were treated with either DMSO or DPE (mg/L). Results are expressed as normalized luciferase activity relative to DMSO (set at 1) (mean ± SEM). Statistical differences are represented by letters.

Pregnane x receptor (PXR), which regulates the metabolism and detoxification of foreign compounds, is known to display species-specific differences with respect to activation by ligands [44], due to sequence differences between mammalian species [86, 87]. We therefore tested both mouse and human PXR. As shown in **Fig 5H**, DPE alone dose-dependently transactivated the mouse PXR chimera, although to a lesser extent than the ligand pregnenolone 16α-carbonitrile (PCN). No significant differences were observed by co-treatment with higher concentrations of DPE and PCN, however, a significant inhibitory effect was seen with 20 mg/L DPE and PCN, compared to PCN alone (**Fig 5H**). In contrast, no such effects were seen with DPE alone and the human PXR chimera and DPE had no effect in the presence of the hPXR ligand, rifampicin (RIF) (**Fig 5I**). The retinoic acid receptor alpha (RARα) and retinoid x receptor (RXR) chimeras were not transactivated by DPE alone, but did both show enhanced transactivation in the presence of their respective ligands and the highest concentration of DPE tested (100 mg/L) (*p*<0.0001) (**Fig 5J and 5K**). No effects were observed for DPE either alone or in combination with their ligands for thyroid hormone receptor beta (TRβ) and vitamin D receptor (VDR) (**Fig 5L and 5M**). Additionally, several other full-length NR-chimera constructs were tested with varying doses of DPE (**Fig 6**), and results show that RORα was modestly transactivated by DPE, while RORβ, mouse and human SHP were unaffected.

**Fig 7:**
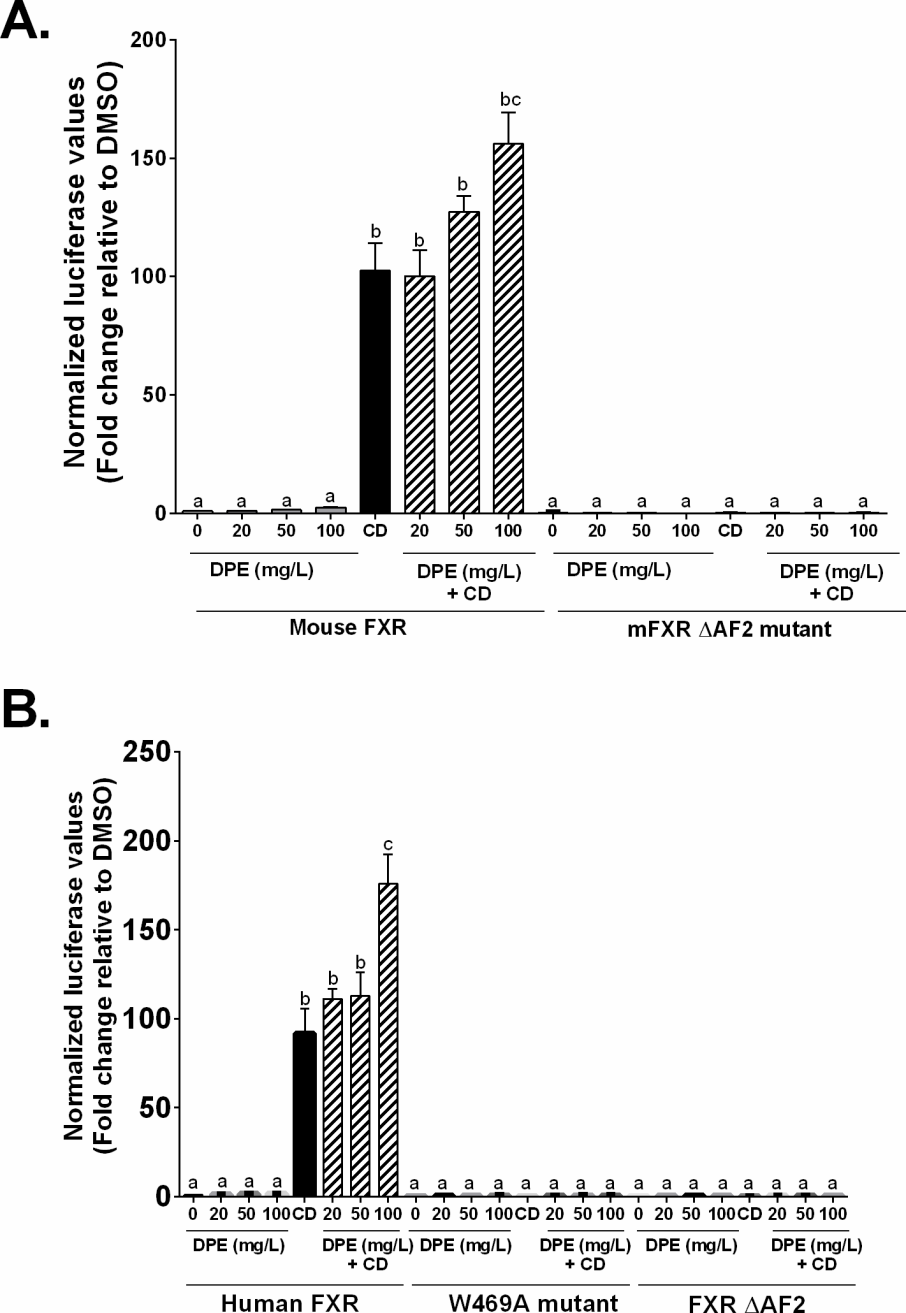
DPE acts as a co-agonist ligand for human and mouse FXR. CV-1 cells were co-transfected with a luciferase reporter construct plus expression vectors as indicated **A.** Mouse FXR and **B.** Human FXR, and treated with vehicle (dimethylsulfoxide, DMSO) (*white bars*), 20, 50 or 100 mg/L DPE (*gray bars*), 100 μM CDCA (*black bars*) or 100 μM CDCA plus DPE as indicated (*hatched bars*). Results are expressed as fold change relative to the control (DMSO), normalized to the β-gal internal control (mean ± SEM). DPE: date palm extract; CD: chenodeoxycholic acid. Statistical differences are represented by letters. Bars with the same superscript letter are not significantly different from each other.

To confirm the co-activation of FXR observed with the Gal4-FXR-LBD chimera, the effect of DPE on the full-length receptors for human and mouse FXR was tested. CDCA activated wild-type mouse and human FXR, but not mutant forms of the receptors, as previously reported [45, 76, 77]. In comparison, DPE alone did not significantly activate human or mouse FXR at the concentrations tested (**Fig 7**). However, there was an enhanced dose-dependent transactivation observed for both the mouse and human receptors in the presence of DPE and CDCA (**Fig 7A and 7B**). For mouse FXR, 50 mg/L DPE + CDCA increased transactivation by 24%, compared to CDCA alone; while 100 mg/L DPE + CDCA increased transactivation by 52%, compared to CDCA alone. In comparison, using human FXR, 50 mg/L DPE + CDCA increased transactivation by 23%, compared to CDCA alone; while 100 mg/L DPE + CDCA increased transactivation by 92%, compared to CDCA alone. These responses were dependent on FXR activation, because they were not observed with the FXR Δ9C mutant, lacking the terminal amino acids 476–484, corresponding to helix 12 in mouse FXR (**Fig 7A**), or the W469A or ΔAF2 human mutant receptor constructs (**Fig 7B**).

**Fig 8:**
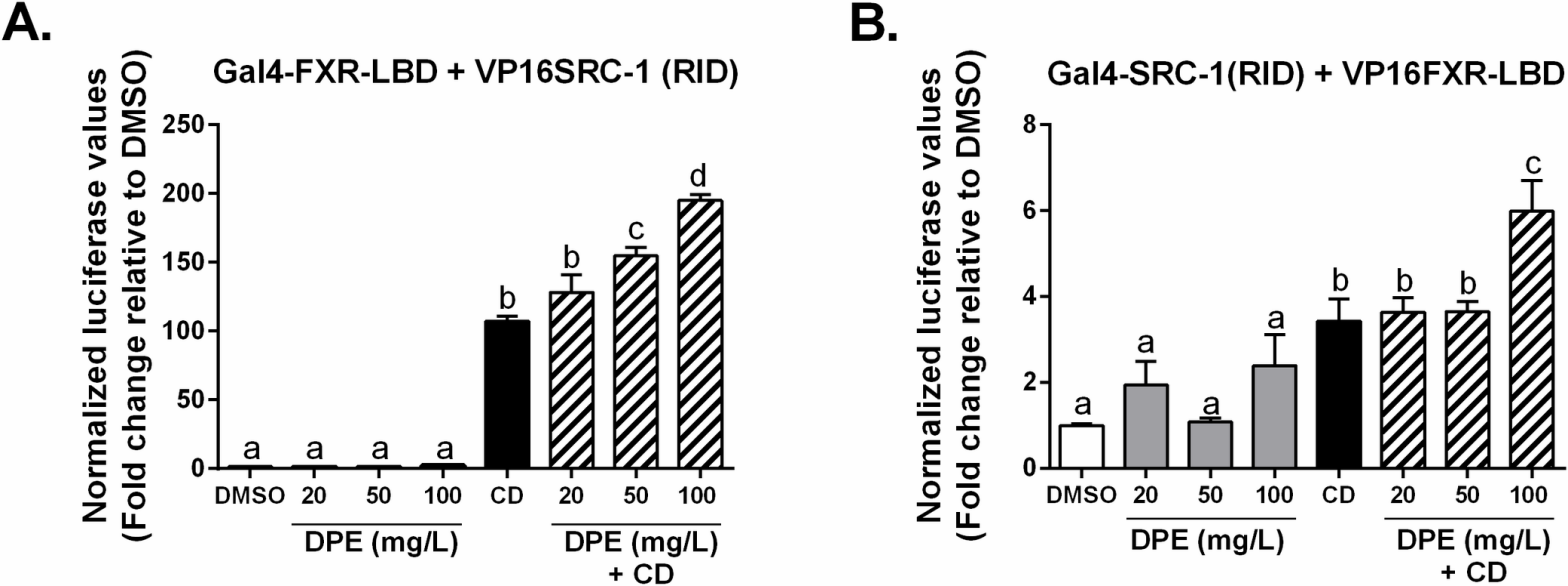
DPE induces coactivator recruitment to bile acid-bound FXR. CV-1 cells were co-transfected with a luciferase reporter construct plus expression vectors as indicated and treated with vehicle (dimethylsulfoxide, DMSO) (*white bars*), 20, 50, or 100 mg/L DPE (*gray bars*), 100 μM CDCA (*black bars*) or 100 μM CDCA + DPE (mg/L) (*hatched bars*). Results are expressed as fold change relative to the control (DMSO), normalized to the β-gal internal control (mean ± SEM). Statistical differences are represented by letters. Bars with the same superscript letter are not significantly different from each other.

A mammalian two-hybrid assay was used to test the ability of DPE to induce coactivator recruitment to mouse FXR. In this assay, firstly, the Gal4-FXR-LBD chimera was tested with the coactivator SRC-1 (steroid receptor coactivator 1) fused to the transactivator VP16, and secondly, with Gal4 fused with the receptor interaction domain (RID) of SRC-1, and mouse FXR fused with VP16 [45]. The ability of CDCA to induce high levels of luciferase expression indicates that it induced interaction of FXR with the coactivator SRC-1 (**Fig 8A and 8B**). The ability of DPE to enhance coactivator recruitment to CDCA-bound FXR is evidenced by the significant increase in transactivation observed compared to CDCA alone (**Fig 8A and 8B**). Overall, these results demonstrate that DPE acts as a co-agonist ligand for FXR and recruits coactivator.

### Date palm extract differentially regulates FXR target-gene expression *in vitro* in Caco-2 cells

After we elucidated that DPE acts as a co-agonist ligand for FXR, we wanted to determine its ability to regulate FXR-target gene expression *in vitro*. The effect of DPE on intestinal FXR-target gene expression was therefore determined using human colorectal Caco-2 cells.

**Fig 9:**
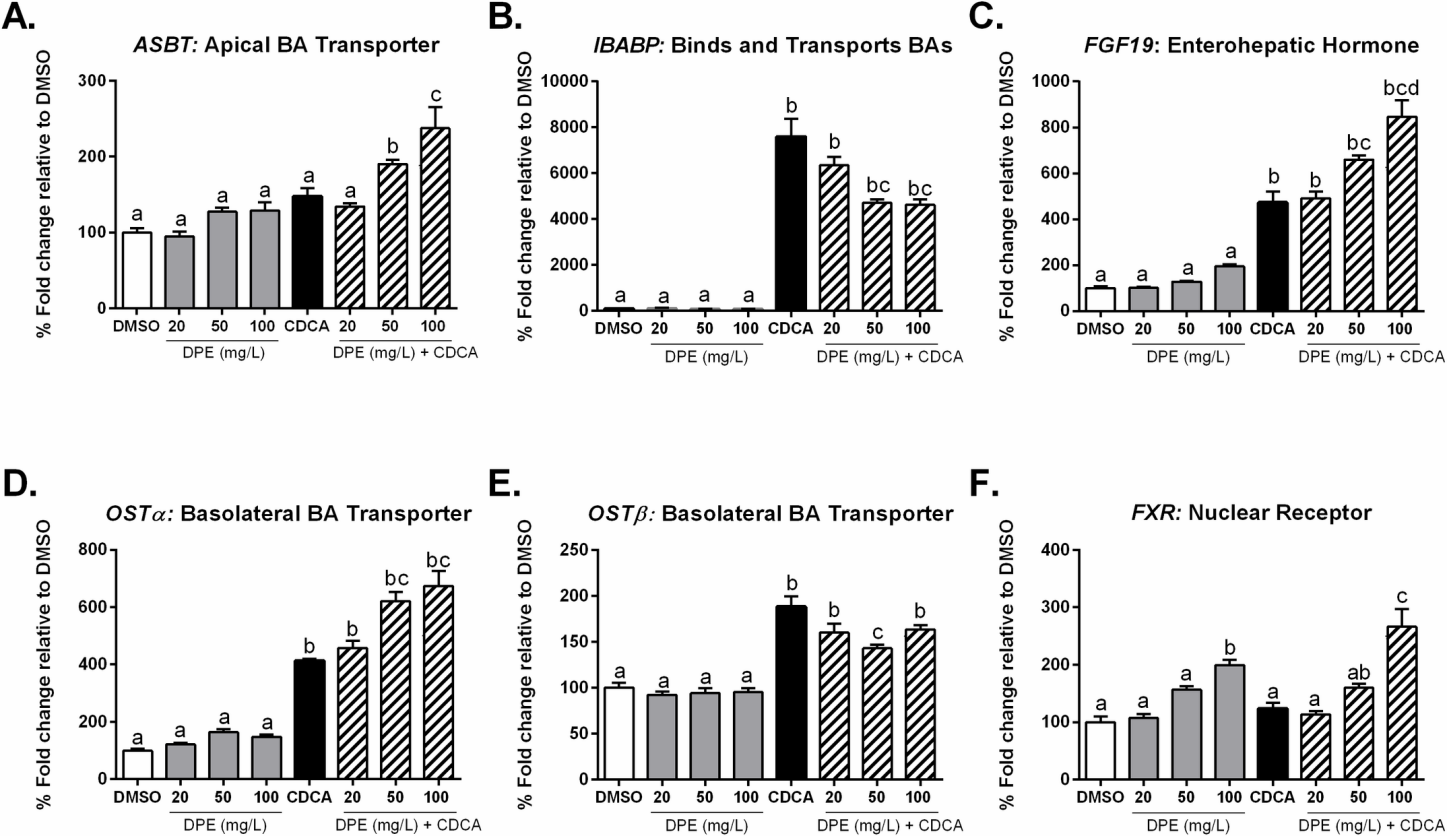
DPE differentially regulates FXR-target gene expression *in vitro* in Caco-2 cells. Caco-2 cells were treated for 12 hours with either a negative control (DMSO), varying doses of DPE (20, 50 or 100 mg/L), 100 μM CDCA, or a combination of CDCA + DPE, as indicated. Relative gene expression is shown for **A.** *ASBT*, **B.** *IBABP*, **C.** *FGF19*, **D.** *OSTα*, **E.** *OSTβ*, and **F.** *FXR*. (Negative control (DMSO), *white bars*; DPE (20, 50 or 100 mg/L) (*gray bars*); CDCA (100 μM) (*black bars*); or in combination (*hatched bars*). Statistical differences are represented by letters. Bars with the same superscript letter are not significantly different from each other.

As shown in **Fig 9A**, after 12 hours treatment, *ASBT* expression was not affected by either CDCA treatment or DPE, but was induced by co-treatment with CDCA + DPE compared to control. As expected, CDCA-treatment increased *IBABP* expression compared to control [42], while co-treatment with DPE dose-dependently inhibited the CDCA-induced increase (**Fig 9B**). No significant differences were observed in *IBABP* expression following treatment with DPE alone. *FGF19* expression (the human homolog to murine *Fgf1*5) was induced by treatment with CDCA, compared to control (**Fig 9C**). Consistent with the notion of DPE as a co-agonist ligand for FXR, co-treatment with CDCA + DPE resulted in a significant dose-dependent increase in *FGF19* expression (**Fig 9C**). Basolateral bile acid transporter expression (*OST*α*/β*) was induced by CDCA, compared to control (**Fig 9D and 9E**), while co-treatment with DPE + CDCA dose-dependently enhanced the CDCA-induced increase in *OSTα* expression (**Fig 9D**). However, co-treatment with DPE led to an inhibition of the CDCA-induced increase in *OSTβ* expression (**Fig 9E**). Although CDCA had no effect on *FXR* expression, DPE did increase expression dose-dependently after 12 hours (**Fig 9F**). Furthermore, co-treatment with CDCA + 100 mg/L DPE significantly increased *FXR* expression after 12 hours (*p*<0.0001) (**Fig 9F**).

## Discussion

The study presented herein demonstrates that California-grown dates are rich in phenolic compounds, including hydroxycinnamic acids, PACs and lipophilic polyphenols, consistent with reports from other date varieties around the world [1, 88, 89]. Furthermore, for the first time we establish that an extract made from date palm fruit acts as a co-agonist ligand for FXR, a nuclear receptor critical for maintaining bile acid, cholesterol, and triglyceride homeostasis [48–54]. Date fruit has served as a staple food in several parts of the world for many centuries and dates have been reported to provide numerous beneficial health effects [89]. For example, a previous report showed that 100 g daily date consumption (equivalent to about 7 dates per day) over a 4 week period reduces serum triglyceride levels in human subjects [25]. However, a detailed mechanistic understanding behind the reported health-related effects has remained unknown. Our novel results strongly suggest that dates could mediate the observed triglyceride lowering effect via FXR.

The presence of phenolic compounds may underlie the reported beneficial health effects of dates. Considerable progress has been made in recent years regarding our knowledge of the bioactive compounds present in plant-based foods and their direct link to human health [89]. Such protective effects have been attributed to the phytochemicals, secondary plant metabolites or integral cellular components, present within fruits [89]. Detailed analysis of the dates used in this study revealed that they are a rich source of phytochemicals, including hydroxycinnamic acids, PACs and lipophilic polyphenols.

Our novel findings show that DPE enhances transactivation of bile acid-bound FXR (**Figs 4 and 7**), as well as recruitment of the co-activator SRC-1 (**Fig 8**), and that it differentially regulates FXR-target gene expression *in vitro* in Caco-2 cells (**Fig 9**). These findings are analogous to the observation that GSPE is a co-agonist ligand for FXR [38] and a naturally occurring *gene-selective* bile acid receptor modulator (BARM) [42]. As we previously described [42], GSPE selectively regulates genes associated with intestinal bile acid absorption and transport via FXR, in which leads to reduced enterohepatic bile acid recirculation and increased bile acid excretion via the feces, leading to reduced serum triglyceride and cholesterol levels [42]. Additional studies have also investigated the potential for synthetic bile acid receptor modulators [90], and other natural compounds to selectively modulate FXR target-gene expression, e.g. the tea catechin, epigallocatechin-3-gallate (EGCG), activates FXR in a tissue- and gene-specific manner [91]. These studies highlight the fact that agonist and co-agonist ligands for FXR can exert complex gene-regulatory effects along the gut-liver axis. For example, CDCA is known to increase *IBABP* expression in order to help maintain bile acid homeostasis [75, 92]. However, analogous to the effects observed with DPE herein, intestinally-mediated effects induced by GSPE include decreased *Ibabp* expression *in vitro* in Caco-2 cells and *in vivo* in C57BL/6 mice, which ultimately contributes to its’ hypotriglyceridemic action [42]. Our *in vitro* results suggest that DPE and date fruit may be a beneficial natural therapy against hypertriglyceridemia due to its ability to differentially modulate FXR target-gene expression in the intestine *in vitro*, and therefore, possibly bile acid absorption and homeostasis *in vivo*.

Unexpectedly, CDCA did not reduce *ASBT* expression at the 12 hour time point tested in this particular study, which is in contrast to previous reports which assessed expression at 24 [42] and 40 hours in Caco-2 cells [79]. Therefore, the lack of an effect by CDCA on *ASBT* expression may relate to the time at which gene expression was analyzed. While *ASBT* expression was not affected by DPE alone, it was increased by co-treatment with CDCA + 100 mg/L DPE.

A limitation of the findings from the current study includes the fact that gene expression analysis was only conducted in cells *in vitro*. Therefore, we cannot definitively predict whether the same gene expression changes will occur *in vivo*, since there are additional factors that would come into play, including signaling effects along the gut-liver axis, as well as the role the gut microbiota may play in the metabolism of phenolic compounds and therefore their absorption into the enterocyte. Based on the characterization data, there is a high degree of polymerization for the polyphenols present within this date extract, which suggests a low bioavailabilty *in vivo*. However, this study does provide evidence to show that date-derived bioactive compounds can traverse the cell membrane and exert gene-regulatory effects within Caco-2 cells, facilitating novel insight into a potential underlying mechanism by which dates could reduce serum triglyceride levels *in vivo*, as observed in the previously reported human study [25]. Further studies are therefore warranted *in vivo* using both wild-type and FXR knockout mouse models to fully determine the extent to which FXR contributes to the molecular mechanism by which dates lower serum triglyceride levels, and such studies are currently on-going.

Although the co-agonistic effects of DPE observed for FXR are consistent with our initial hypothesis, we also observed an agonistic action for DPE with the PPARα-LBD chimera (**Fig 5F**). PPARα is a nuclear receptor essential for increasing triglyceride catabolism via enhanced fatty acid β-oxidation. Previous studies identified GSPE as an HDAC inhibitor leading to enhanced PPARα gene transcription, increased PPARα protein phosphorylation and increased PPARα target-gene transcription, correlating with modulated lipid catabolism and reduced serum triglycerides *in vivo* [40]. Although GSPE acts as an HDAC inhibitor, when tested, DPE did not affect HDAC activity (Ferguson and Ricketts, unpublished observations), further supporting the conclusion from the transient transfection studies that DPE acts as an agonist ligand for PPARα. Further studies are therefore warranted to fully determine the effects that could be mediated by DPE via PPARα.

Screening a series of Gal4-NR chimera constructs facilitated determination of the specificity of DPE for FXR. The results show that in addition to PPARα, PPARϒ was modestly transactivated (3-fold, p<0.05) by DPE at the highest concentration tested (100 mg/L) (**Fig 5G**). Although mouse PXR was dose-dependently transactivated by DPE (**Fig 5H**), it is important to note that human PXR displayed no such effect (**Fig 5I**). Transactivation for the Gal4-GR-LBD chimera was significantly and dose-dependently enhanced by co-treatment with DPE and dexamethasone (**Fig 5D**), which may warrant further investigation. Additionally, transactivation for both RARα and RXR chimeras were enhanced by co-incubation with both 100 mg/L DPE and their respective ligands, but not at the lower concentrations tested, while no effects were seen with the chimeras for mCAR, hCAR, ERα, LXRα, TRβ and VDR. It remains unknown at this time if any of the above mentioned effects would also be observed with the full-length receptors and whether they would lead to any potential physiological consequences, therefore further studies may be needed.

Although dates contain 70–80% sugars it is important to note, however, that they have a low glycemic index (42 ± 4) [93]. The glycemic index of a particular food depends on the rate of digestion and absorption of its carbohydrate content, such that a glycemic index of <55 is considered low, 56–59 is considered medium, while a glycemic index of >70 is considered high [94]. This is particularly relevant to diabetic subjects who often receive conflicting advice regarding date consumption. A previous report showed that consumption of 50 g of available carbohydrates from 5 different varieties of dates by diabetic subjects did not lead to a significant rise in post-prandial glucose levels [95]. It should be noted, however, that the diabetic subjects who participated in the study used either diet or metformin to maintain their glucose levels and none were receiving insulin or multiple oral hypoglycemic agents [95]. Furthermore, no subjects with type 1 diabetes were included in the study. Additional studies may therefore be warranted to confirm whether a lack of effect on post-prandial glucose levels is also observed in type 1 diabetic subjects or those with type 2 diabetes who use insulin and whether this effect holds true for all varieties of date palm fruit.

In conclusion, this study shows that dates contain bioactive compounds which exert FXR-mediated regulatory effects that may contribute to the underlying molecular mechanism involved in the triglyceride-lowering action of dates. Additionally, this study identifies a new potential intestinally-mediated mechanism by which poorly-bioavailable polyphenols from dates could affect blood lipid levels without being absorbed systemically. Further research is therefore warranted to enhance our understanding of a role for FXR and the potential for date polyphenols to contribute to the amelioration of human metabolic dysregulation and disease.

## Abbreviations

CDCA: Chenodeoxycholic acid
DPE: Date palm extract
HPLC-DAD: High performance liquid chromatography with diode-array detector
LBD: Ligand binding domain
MALDI-TOF-MS: Matrix-assisted laser desorption-ionization time-of-flight mass spectrometry
MetS: Metabolic syndrome
NR: Nuclear receptor
